# Systematic analysis of Type I‐E *Escherichia coli* CRISPR‐Cas PAM sequences ability to promote interference and primed adaptation

**DOI:** 10.1111/mmi.14237

**Published:** 2019-04-06

**Authors:** Olga Musharova, Vasily Sitnik, Marnix Vlot, Ekaterina Savitskaya, Kirill A. Datsenko, Andrey Krivoy, Ivan Fedorov, Ekaterina Semenova, Stan J. J. Brouns, Konstantin Severinov

**Affiliations:** ^1^ Center of Life Sciences Skolkovo Institute of Science and Technology Moscow 121205 Russia; ^2^ Institute of Molecular Genetics Russian Academy of Sciences Moscow 123182 Russia; ^3^ Laboratory of Microbiology, Department of Agrotechnology and Food Sciences Wageningen University Stippeneng 4 Wageningen 6708 WE The Netherlands; ^4^ Department of Biological Sciences Purdue University West Lafayette IN 47907 USA; ^5^ Department of Bionanoscience Kavli Institute of Nanoscience, Delft University of Technology Van der Maasweg 9 Delft 2629 HZ The Netherlands; ^6^ Waksman Institute Rutgers, The State University of New Jersey Piscataway NJ 08854 USA

## Abstract

CRISPR interference occurs when a protospacer recognized by the CRISPR RNA is destroyed by Cas effectors. In Type I CRISPR‐Cas systems, protospacer recognition can lead to «primed adaptation» – acquisition of new spacers from *in cis* located sequences. Type I CRISPR‐Cas systems require the presence of a trinucleotide protospacer adjacent motif (PAM) for efficient interference. Here, we investigated the ability of each of 64 possible trinucleotides located at the PAM position to induce CRISPR interference and primed adaptation by the *Escherichia coli* Type I‐E CRISPR‐Cas system. We observed clear separation of PAM variants into three groups: those unable to cause interference, those that support rapid interference and those that lead to reduced interference that occurs over extended periods of time. PAM variants unable to support interference also did not support primed adaptation; those that supported rapid interference led to no or low levels of adaptation, while those that caused attenuated levels of interference consistently led to highest levels of adaptation. The results suggest that primed adaptation is fueled by the products of CRISPR interference. Extended over time interference with targets containing «attenuated» PAM variants provides a continuous source of new spacers leading to high overall level of spacer acquisition.

## Graphical abstract

All possible of 64 PAM combinations were tested. 36 trinucleotides were completely unable to support interference. The remaining 28 PAM trinucleotides fell into two groups: those that supported fast interference and those that supported intermediate, delayed interference that was apparently countered by plasmid copy number maintenance mechanisms over extended periods of time. PAM variants that did not lead to CRISPR interference also did not support primed adaptation. PAM variants supporting intermediate‐rate interference caused strong priming.

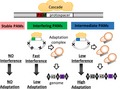

## Introduction

CRISPR‐Cas (Clustered Regularly Interspaced Short Palindromic Repeats‐CRISPR associated genes) systems provide prokaryotes with resistance against mobile genetic elements (MGEs), such as plasmids and bacteriophages (Barrangou *et al.*, 2007; Brouns *et al.*, 2008; Marraffini and Sontheimer, 2008). While highly diverse, all CRISPR‐Cas systems share a common defensive strategy and operate through three stages: adaptation, expression and interference (Makarova *et al.*, 2015; Shmakov *et al.*, 2017). During adaptation, invader DNA sequences called protospacers are incorporated into CRISPR arrays as spacers (van der Oost *et al.*, 2009). The process of spacer acquisition is mediated by the evolutionarily conserved Cas1 and Cas2 proteins (Makarova *et al.*, 2006; Yosef *et al.*, 2012; Nuñez *et al.*, 2015; Koonin *et al.*, 2017; Wright *et al.*, 2017). During the expression stage, CRISPR arrays are transcribed into pre‐CRISPR RNA and further processed into short CRISPR RNAs (crRNAs). At the interference stage, individual crRNAs bind to Cas proteins and the resulting effector complexes recognize protospacers complementary to crRNA spacer segments (Brouns *et al.*, 2008; Jore *et al.*, 2011; Wiedenheft *et al.*, 2011; Szczelkun *et al.*, 2014). This recognition ultimately leads to destruction of protospacer‐containing DNA. In Type I CRISPR‐Cas systems, degradation is performed by the Cas3 helicase–nuclease (Mulepati and Bailey, 2013; Hochstrasser *et al.*, 2014; Gong *et al.*, 2014; Jackson *et al.*, 2014; Künne *et al.*, 2016). To avoid autoimmunity caused by the recognition of spacers in CRISPR arrays, Type I systems rely on the presence of an additional element, the ‘protospacer adjacent motif’ or PAM, located at the 5′ flank of protospacers (Deveau *et al.*, 2008; Mojica *et al.*, 2009). The corresponding position of a CRISPR repeat is distinct from the PAM, which prevents self‐recognition (Westra *et al.*, 2013; Xue *et al.*, 2015).

The Cas1 and Cas2 proteins are capable of *de novo* acquisition of spacers into a CRISPR array. However, this process is inefficient. First, it does not exclusively target foreign DNA leading to acquisition of self‐targeting spacers from cell’s own genome. Second, at least half of the spacers are selected from protospacers with dysfunctional PAMs (Yosef *et al.*, 2012; Díez‐Villaseñor *et al.*, 2013; Levy *et al.*, 2015; Bozic *et al.*, 2019) and resulting crRNAs are not interference‐proficient.

Even if a spacer targeting a foreign protospacer with an interference‐proficient PAM is acquired, MGEs can rapidly evolve resistance to CRISPR interference by acquiring mutations in the PAM or the protospacer, preventing or decreasing the efficiency of recognition by the effector. A process named ‘primed adaptation’ described for various Type I CRISPR‐Cas systems allows the cell to effectively counter MGEs that escape CRISPR interference (Datsenko *et al.*, 2012; Fineran and Charpentier, 2012; Swarts *et al.*, 2012; Sternberg *et al.*, 2016; Jackson *et al.*, 2017). Primed adaptation leads to highly efficient and targeted accumulation of new spacers located *in cis* to the ‘priming’ protospacer recognized by the effector (Savitskaya *et al.*, 2013). The apparent yields of primed adaptation (measured as the number of extended CRISPR arrays in the population) are very low if a target protospacer is fully matched with crRNA and contains a consensus interference‐proficient PAM (AAG or ATG in the case of *E. coli* I‐E system) (Datsenko *et al.*, 2012; Xue *et al.*, 2015; Wang *et al.*, 2015; Semenova *et al.*, 2016). Primed adaptation yield is stimulated by the presence of PAM or protospacer mutations that decrease the interference efficiency (Semenova *et al.*, 2011; Jinek *et al.*, 2012; Richter *et al.*, 2014; Severinov *et al.*, 2016). Yet, primed adaptation requires the functional Cas3 protein, suggesting a functional link between CRISPR interference and primed adaptation (Fineran *et al.*, 2014; Künne *et al.*, 2016; Dillard *et al.*, 2018). Two alternative models have been put forward to explain such a link. One model posits that effectors bound to protospacers with certain PAMs assume a specific conformation that recruits the adaptation machinery (Cas1 and Cas2) as well as the Cas3 protein, followed by directional scanning of the target and selection of new spacers (Redding *et al.*, 2015). In contrast, complexes formed on targets with interference‐proficient PAMs do not support Cas1–Cas2 recruitment, leading to interference only (Sashital *et al.*, 2012). The second model postulates that apparent difference in primed adaptation yield with various targets is a consequence of dynamics of degradation of less‐than‐optimal targets (Künne *et al.*, 2016; Semenova *et al.*, 2016). Since most MGEs are able to replicate and have copy‐number maintenance mechanisms of their own, the competition between attenuated CRISPR interference and copy number maintenance mechanisms may create a situation when degradation fragments of MGE genomes are present in the cell for extended time, allowing the presumably slower adaptation reaction to occur (Severinov *et al.*, 2016). To systematically investigate the connection between CRISPR interference and primed adaptation for *E. coli* I‐E system, here we directly compared the ability of all of the 64 possible trinucleotides installed in the position of PAM to support interference and primed adaptation. While broadly supporting the kinetic model predictions, we find several PAM variants whose behavior is apparently consistent with specific adaptation‐prone conformation of the priming complex.

## Results

### Using a comprehensive PAM library to study CRISPR interference *in vivo*

**Figure 1:**
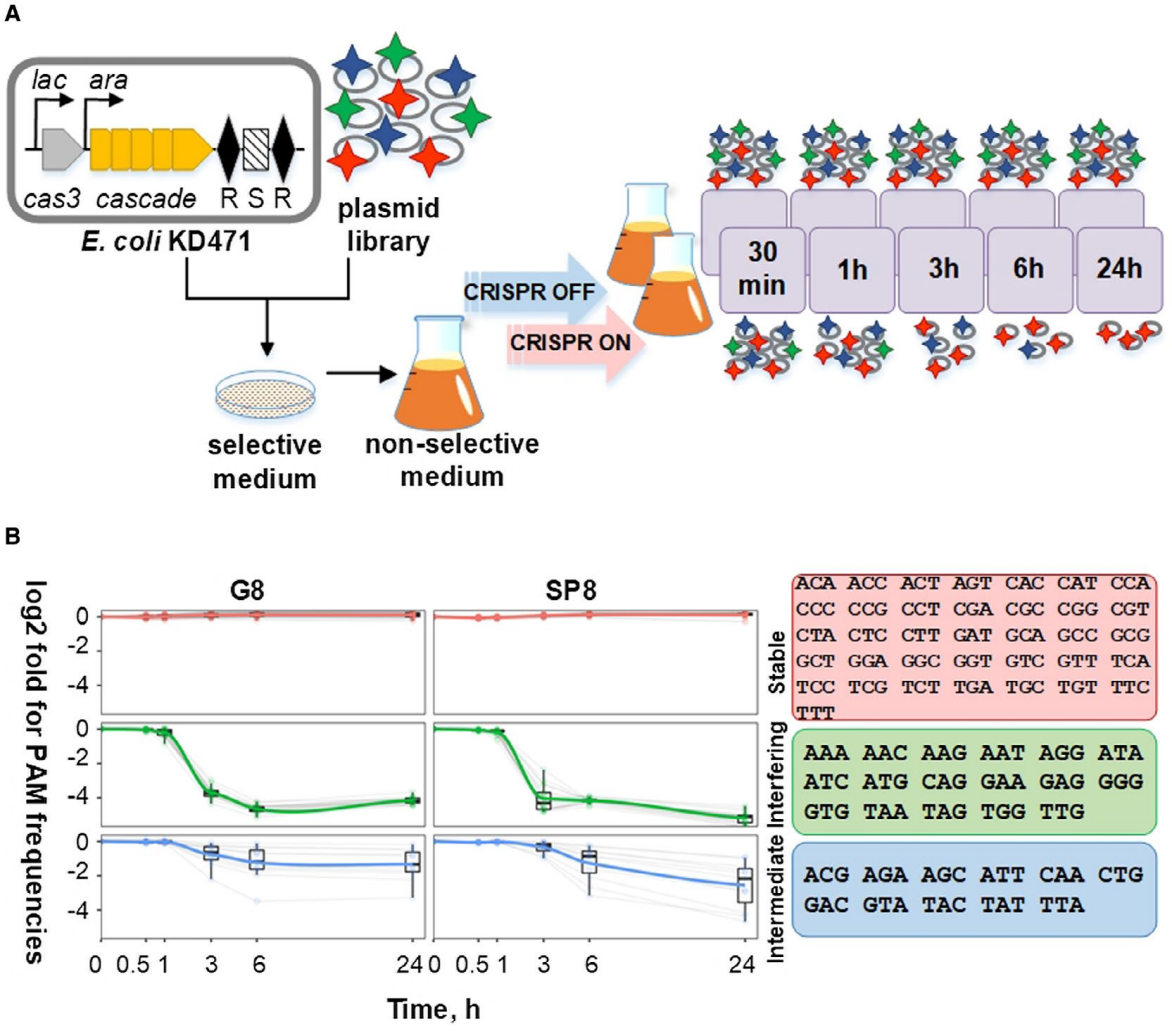
High‐throughput analysis of PAM sequences effect on CRISPR interference. A. On the top, an engineered *E. coli* cells with inducible expression of *cas* genes coding for Type I‐E interference machinery and lacking the *cas1* and *cas2* genes coding for the adaptation enzymes are schematically shown. The cells contain a CRISPR array with a single spacer (S) located between two repeats (R). The workflow of the PAM library experiment is presented. Cells are transformed with a library of plasmids containing a protospacer matching CRISPR array spacer and randomized trinucleotide at the PAM position (shown by colored stars). Transformants grown on selective medium are pooled and placed in nonselective medium without antibiotic required for plasmid maintenance. The cultures are divided and allowed to grow in the presence (CRISPR ON) on in the absence (CRISPR OFF) of *cas* gene expression inducers. At various time points, culture aliquots are removed, plasmid purified and the frequency of remaining PAM variants determined. B. Frequency change of PAM variants in SP8 and G8 protospacer plasmid libraries in induced cells over time. Based on their behavior, PAM variants are divided into stable, interfering and intermediate group. The error bars indicate the extent of variation observed for individual members within each group. Box plots show the range of frequencies for 75% of group members. Individual PAMs belonging to each group are listed at the right hand side of the figure. [Colour figure can be viewed at https://wileyonlinelibrary.com]

To determine the ability of every possible trinucleotide to function as a PAM during *E. coli* Type I‐E CRISPR interference, we prepared two plasmid libraries each containing a previously characterized protospacer – SP8 (Swarts *et al.*, 2012) or G8 (Semenova *et al.*, 2011) – and a randomized upstream trinucleotide. Two specially designed *E. coli* strains (Fig. 1A, top) were used to transform plasmid libraries. In both strains, expression of *cas* genes can be induced by the addition of arabinose and IPTG. Both strains contain a single miniature CRISPR array with just one spacer – G8 for KD471 and SP8 for KD635. To monitor CRISPR interference effects without secondary contributions by spacers acquired during primed adaptation, the *cas1* and *cas2* genes in KD471 and KD635 were deleted.

To assess CRISPR interference against protospacers with various PAMs, a pooled plasmid loss experiment was performed (Fineran *et al.*, 2014). The workflow of the experiment is shown in Fig. 1A. Thousands of antibiotic‐resistant colonies obtained after transformation of each plasmid library into uninduced cognate cells were pooled and resuspended in a medium without antibiotic. Half of the culture was induced to initiate expression of *cas* genes (‘CRISPR ON’), while the other half was uninduced (‘CRISPR OFF’) and served as a control. The cultures were allowed to grow and at various times aliquots were withdrawn and plasmid DNA was isolated. The region containing the target protospacer was PCR amplified and sequences of amplified fragments were determined by high‐throughout sequencing (HTS) using an Illumina platform. After sequencing, the abundance of reads corresponding to each PAM variant was determined and normalized for sequencing depth. In CRISPR OFF cultures, the relative frequencies of individual PAM variants remained unchanged with time, as expected. In contrast, in CRISPR ON cultures, frequencies of individual variants changed dramatically in the course of culture growth, presumably due to CRISPR interference. As some library variants became lost during growth, the relative frequency of remaining ones increased. A normalization procedure described in the Materials and Methods section allowed us to compensate for this effect. In Fig. 1B, the results of analysis performed with both protospacer libraries are summarized. As can be seen, different PAM sequences clearly separated into three groups. Variants whose relative ratios started to strongly decrease 1.5 h after induction and that reached stable very low levels at 3 h were considered as ‘interfering’. The 17 interfering variants included the consensus ATG and AAG PAMs. The interfering groups were identical in the G8 and SP8 libraries (Spearman coefficient 0.809). Another group (36 variants) named ‘stable’ included sequences whose relative ratios remained unchanged after growth at inducing conditions. Again, this group consisted of identical members in the SP8 and G8 libraries (Spearman coefficient 0.89) and contained the CCG sequence found in the CRISPR repeat. The final group, which we refer to as ‘intermediate’, contained sequences whose relative ratios decreased over time, however, the kinetics of decay was much slower than in the interfering group: the ratios of intermediate sequences remained stable 1.5 h post‐induction and slowly decreased at later times. Compared to the ‘interfering’ and ‘stable’ group members, the individual behavior of 11 variants in the intermediate group was more diverse, both within and between libraries (Spearman coefficient 0.414). In particular, the relative ratio of the intermediate AGA variant approached, at later times, levels comparable to those of ‘interfering’ PAMs in both libraries.

**Figure 2:**
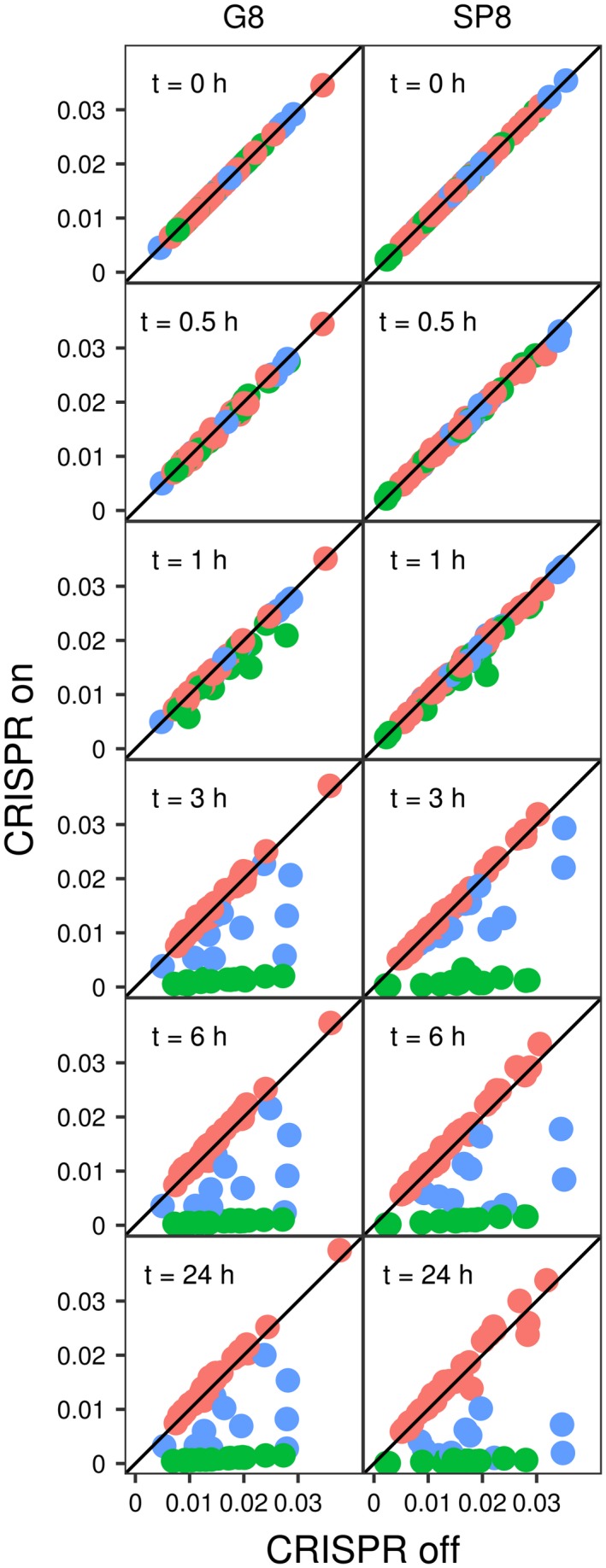
Dynamics of frequency change of SP8 and G8 protospacer plasmids with different PAM variants. Each dot represents the frequency of individual PAM variant plasmid frequency under CRISPR ON (vertical axis) and CRISPR OFF (horizontal axis) conditions at various times. Red dots indicate PAM variants belonging to the stable group, green – Interfering, and blue – intermediate group PAMs. For each panel, the frequency of dots located on black diagonal is the same in CRISPR ON and CRISPR OFF conditions. The dynamics of PAM variant behavior is also shown in an animation on Supporting Fig. S2B. Note that every dot remains in its group (does not change color) with time. [Colour figure can be viewed at https://wileyonlinelibrary.com]

The temporal dynamics of all PAM variants in each library is shown in Fig. 2, where changes of relative frequencies of each PAM in the CRISPR OFF and CRISPR ON libraries over time are illustrated. Dots indicating individual variants start off on a diagonal at time 0. By 1 h, the interfering variants (green dots) start to move leftward (decreased frequency in CRISPR ON library) and reach stable low numbers at the 3‐h time point. Stable variants (red dots) remain on the diagonal even after 24 h of incubation at inducing conditions. The intermediate variants (blue dots) form a diverse group that over the course of the experiment ‘travels’ in the area bounded by the interfering and stable variants, with some members approaching the former group late in experiment. An animation presented in Supporting Fig. S2 illustrates this behavior. An additional animation, in Supporting Fig. S2, allows one to follow the behavior of each individual PAM variant separately.

### Determining the ability of individual PAMs to cause primed adaptation

**Figure 3:**
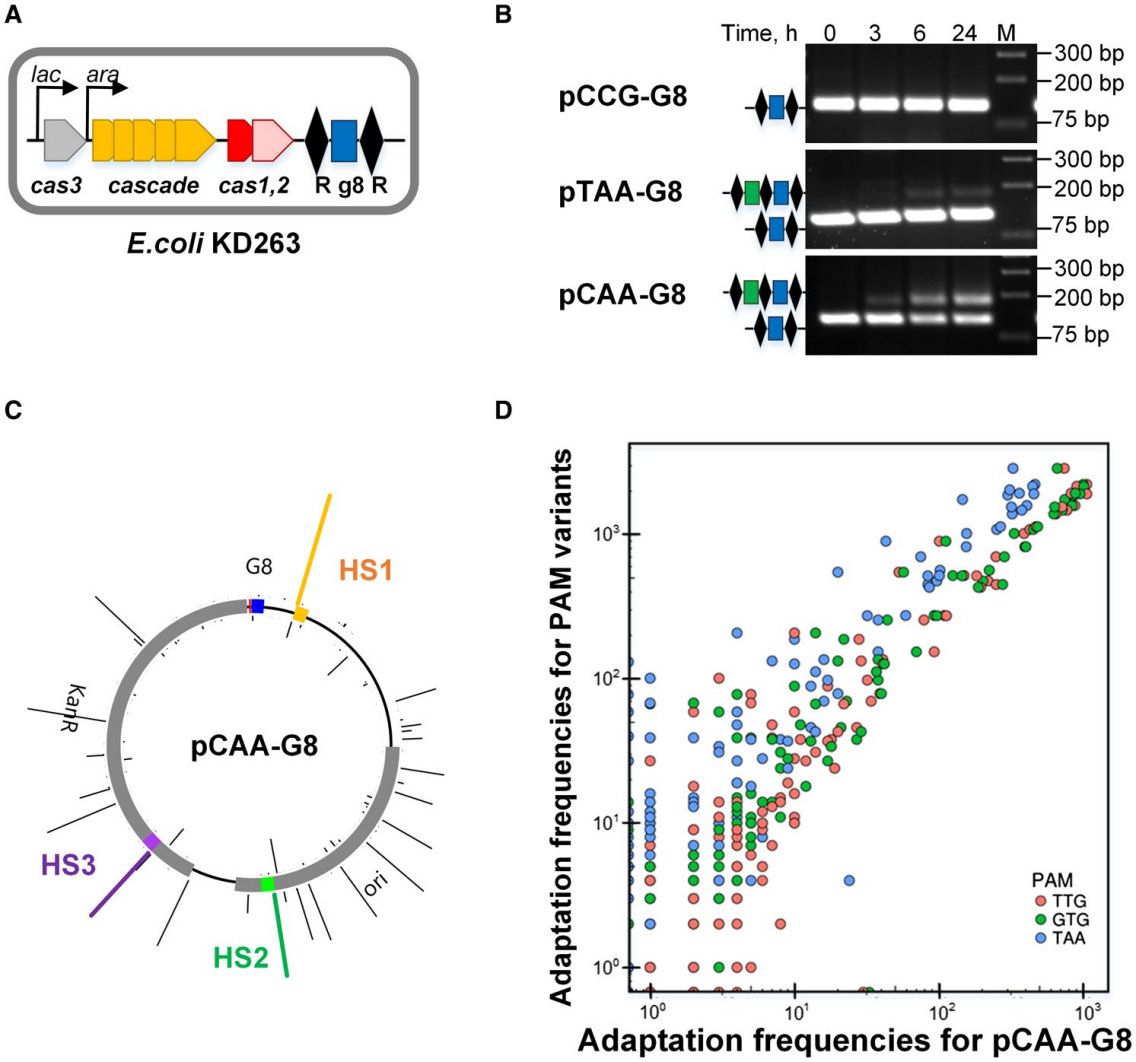
*In vivo* analysis of adaptation from plasmids containing priming protospacers with selected PAM variants. A. *E. coli* KD263 cell containing inducible *cas* genes required for both interference and adaptation and a CRISPR array with a single G8 spacer located between two repeats (R) is shown. B. The results of PCR analysis of induced KD263 cultures harboring G8 protospacer plasmids with CCG, TAA and CAA PAM variants is presented. At indicated times post‐induction CRISPR arrays were amplified and resolved by agarose gel electrophoresis. Bands corresponding to initial and expanded CRISPR arrays are shown. C. Mapping of spacers acquired from the pCAA‐G8 PAM variant plasmid to the pT7blue‐Km backbone (see Supporting Fig. S3A for other PAM variant plasmids). The height of the bars corresponds to the number of HTS reads found for a particular position. The location of the priming G8 protospacer and its PAM is shown (blue and red, correspondingly). Hotspots HS1, HS2 and HS3 which were used for qPCR analysis are marked orange, green and purple respectively. D. Position‐dependent acquisition frequency of spacers in CRISPR arrays of cells carrying plasmids with TTG, GTG and TAA PAM variants by the G8 protospacer is plotted over the acquisition frequency observed in cells harboring a plasmid with the CAA PAM. [Colour figure can be viewed at https://wileyonlinelibrary.com]

The high‐throughput/library approach does not allow one to examine the ability of individual PAM variants to induce primed adaptation since plasmid backbones from which new spacers are acquired are identical in every library member. Therefore, we assembled a collection of 64 individual plasmids carrying the G8 protospacer and each of the possible trinucleotide in the place of PAM. Each plasmid was transformed into KD263 strain (Shmakov *et al.*, 2014), which is isogenic to KD471 but carries functional *cas1* and *cas2* genes (Fig. 3A). After transformation, cultures were induced in liquid medium, and at various times post‐induction the region of genomic DNA containing the CRISPR array was amplified and amplification products were resolved by agarose gel electrophoresis. Characteristic patterns observed in such experiments are shown in Fig. 3B. In some cultures, only the ~120 bp amplicon corresponding to unexpanded KD263 array was detected (the CCG ‘repeat’ variant as well as all other members of the ‘stable’ group, above, exhibited such behavior). Other cultures displayed at least some level of adaptation, which was revealed by the appearance of a ~180 bp amplicon corresponding to CRISPR array expanded by one spacer‐repeat unit. The yield of expanded array amplicon and the time of its appearance differed significantly for cultures harboring plasmids with different PAM variants (see examples of TAA and CAA variants in Fig. 3, and Supporting Fig. S3).

HTS analysis of spacers acquired from plasmids with four randomly chosen variants of PAM, CAA, TTG, GTG and TAA, showed that the overall usage of potential protospacers as sources of new spacers was highly correlated between the plasmids (Pearson coefficients above 0.95, Fig. 3D). We also analyzed spacer acquisition from several PAM variant plasmids containing the SP8 protospacer (Supporting Fig. S5B–D). The results showed that the pattern of spacer acquisition was highly correlated between different SP8 plasmids and spacers acquired from plasmids containing the G8 and SP8 priming protospacers were also correlated, indicating that the nature of the priming spacer has no effect on the choice of new spacers acquired.

The resolution and sensitivity of the gel‐based assay is not sufficient to reveal quantitative information about primed adaptation efficiency caused by different PAM variants. Therefore, we used a more sensitive and quantitative qPCR‐based procedure (Krivoy *et al.*, 2018). The procedure relies on detecting amplicons obtained with a pair of primers, one specific to the CRISPR array leader and another complementary to hot spot (HS) plasmid‐derived spacer that is efficiently acquired during primed adaptation. Using defined mixtures of cells with unexpanded array and cells containing an extra spacer complementary to the primer used for qPCR, a calibration curve can be built to determine an ‘adaptation score’, i.e. the percentage of cells that acquired the spacer (Supporting Fig. S4E‐G). We monitored primed adaptation in cultures of cells transformed with individual plasmids containing different PAM variants in front of the G8 protospacer with primers specific to three hot spots, HS1, HS2 and HS3, each accounting for 5%–8% of total acquired spacers.

**Figure 4:**
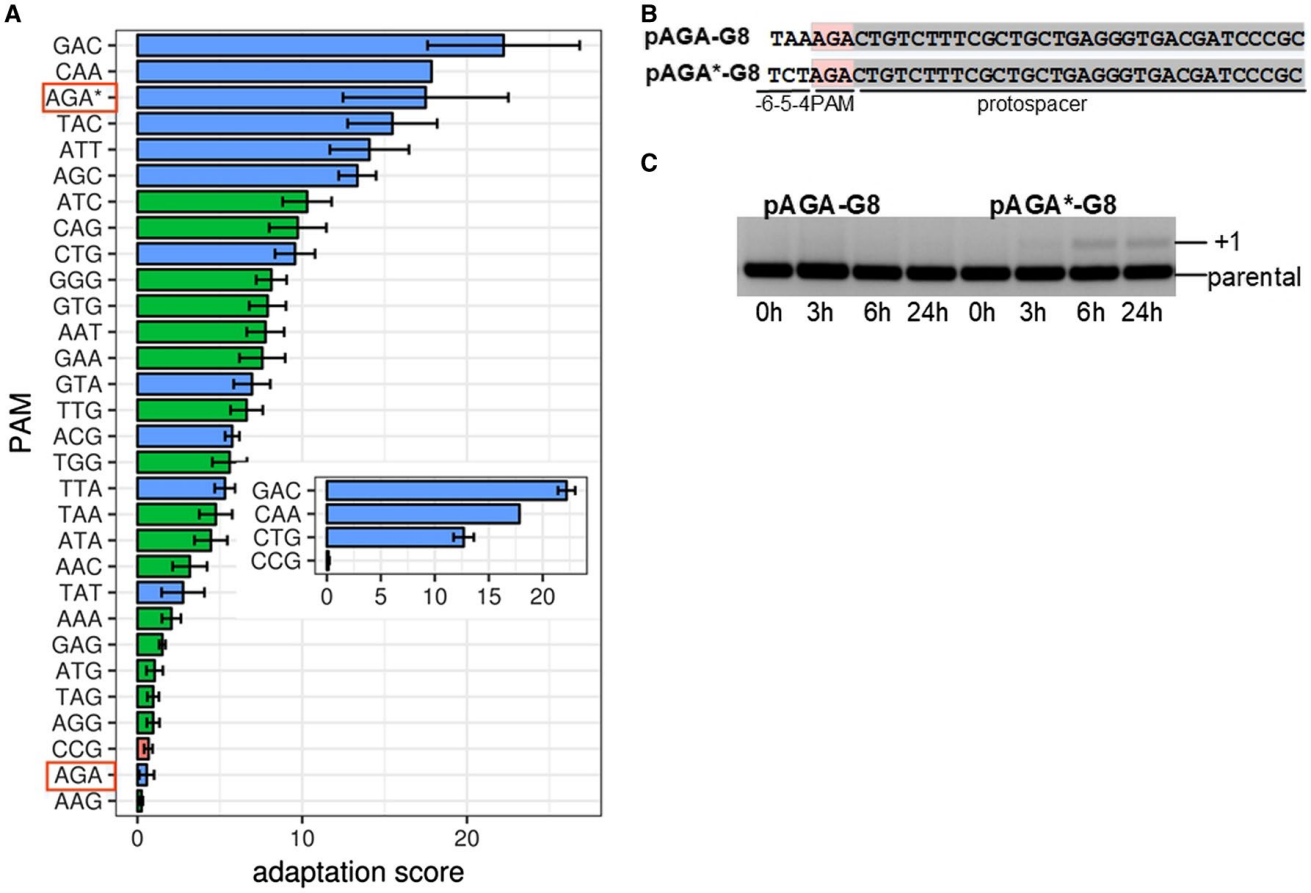
Quantitative measurements of adaptation efficiency from priming protospacer with interfering and intermediate PAMs. A. The adaptation score (see Experimental procedures) or priming from G8 protospacer associated with different PAM variants. Mean values obtained from three independent measurements are shown along with standard deviations. Bars colored blue indicate intermediate group PAMs; green bars shown interfering PAMs. Red bar shows the result obtained with CCG PAM belonging to the stable group. The adaptation score of this variant is the same as that obtained with mock control (water instead of cell culture). Adaptation scores obtained with AGA PAM in the initial and changed context (panel B) are highlighted with red boxes. The adaptation score in changed context is additionally indicated by an asterisk. In the inset, the adaptation scores obtained with cells carrying several SP8 protospacer plasmids with indicated PAM variants are shown. B. Above, the sequence of G8 protospacer associated with the AGA PAM variant and upstream sequence in the plasmid from initial library is shown. Below, the sequence in a modified plasmid removing the off‐set AAG trinucleotide present in the initial sequence is presented. C. Dynamics of CRISPR arrays expansion in cells transformed with AGA PAM variant plasmids shown in B is presented. An agarose gel is shown. [Colour figure can be viewed at https://wileyonlinelibrary.com]

Using the calibration curves presented in Supporting Fig. S4E–G, we calculated adaptation scores 6‐h post‐induction for cells carrying 28 PAM plasmid variants from the interfering and intermediate groups. As a negative control, a single stable group PAM variant plasmid, CCG, was included. The adaptation score was determined in three independent experiments for HS1, HS2 and HS3 for each of the PAM variant plasmid. As expected, the adaptations score of ~0 was observed for each of the three hot spots in cultures transformed with the CCG ‘repeat’ PAM variant plasmid (Fig. 4A). In cases when adaptation was detected, PAMs that led to highest adaptation scores belonged to the intermediate group, while PAMs with lowest adaptation scores belonged to the interfering group (Fig. 4A). The relative adaptation efficiency appeared to depend on the PAM, rather than the protospacer sequence, since the adaptation score of several randomly selected PAM variants was the same with both G8 and the SP8 protospacer (Fig. 4A, inset). In particular, the GAC trinucleotide led to the highest adaptation levels with both protospacers.

One notable exception from the ‘intermediate interference results in higher adaptation score’ rule was the AGA PAM, which caused very poor adaptation. As can be seen from Fig. 4B, a dinucleotide immediately adjacent to randomized PAMs in our library is 5′‐AA‐3′. Thus, for AGA, as well as AGC, AGT and AGG PAM variants, an upstream AAG consensus interference PAM is present. While this PAM is off‐register with respect to protospacer recognized by crRNA, it is possible that it might be recognized by the Cse1 subunit due to its conformational mobility (Xue *et al.*, 2016) and/or lateral diffusion of the Cascade effector complex on priming protospacer, thus affecting target recognition, target destruction and primed adaptation yield. To test this conjecture, the G8 protospacer with four AGN PAM variants was recloned into a background that contained upstream 5′‐CT‐3′ sequence instead of the initial 5′‐AA‐3′. The new constructs contain a TAG trinucleotide instead of the putative off‐register AAG PAM. Plasmids containing AGN PAM variants were individually analyzed for their ability to induce primed adaptation. The AGT and AGG variants, which belonged, respectively, to stable and interfering groups in the initial library and both had poor adaptation scores, continued to be unable to induce adaptation in the new background. The AGC variant originally scored as intermediate and adaptation‐proficient also retained these properties in the new background. In contrast, the AGA PAM, which adapted poorly in the original context (see above) became highly proficient in adaptation (Fig. 4C, Supporting Fig. S6B). Further, this variant, which was the most strongly interfered with member of the intermediate group in the original background, became considerably more stable, i.e. was better able to withstand interference. We consider these results as evidence that an off‐register AAG PAM can induce interference and, as a consequence affect adaptation efficiency of correctly positioned PAMs it overlaps with. The result also establishes that the AGA PAM, an outlier in the original library analysis, in fact conforms to the ‘slow interference‐high adaptation’ rule.

## Discussion

Protospacer adjacent motifs allow Type I and Type II systems to differentiate self from non‐self DNA during CRISPR interference. In the case of *E. coli* Type I‐E system, consensus PAM derived from early bioinformatics analysis (Mojica *et al.*, 2009) and experimental data (29, 45, 46) (Fineran *et al.*, 2014; Xue *et al.*, 2015; Leenay *et al.*, 2016) of viral and plasmid protospacers targeted by spacers present in CRISPR array is either ATG or AAG. Our earlier limited analysis of 26 PAM variants located upstream of G8 protospacer revealed that multiple additional PAMs can also support interference at least as well as the consensus PAMs (Westra *et al.*, 2013). More recently, Fu *et al. *(2017) performed a functional screen by screening *E. coli* cells harboring a library of crRNAs recognizing phage *λ* genome for ability to withstand phage infection. By design, such an approach identified PAM sequences that function with different protospacers. 22 trinucleotides associated with protospacers that allowed protection of cells from phage infection were identified, while 42 trinucleotides were associated with protospacers that were unable to protect from infection and were therefore nonfunctional.

We here systematically screened all 64 trinucleotides for ability to support CRISPR interference when located at PAM position of two different protospacers. The results obtained are identical for both protospacers, which suggests that for a given spacer–protospacer pair, the identity of PAM is the primary determinant of interference efficiency. The Cse1 subunit of the Cascade effector that, based on structural data, is specifically recognizing the AAG consensus PAM sequence (Hayes *et al.*, 2016) and must be responsible for observed relative efficiencies of various trinucleotides ability to serve as functional PAM. However, since Cse1 recognizes PAM through the minor grove, the recognition is inherently promiscuous as is indeed observed in our analysis.

Overall, considering the differences in approaches used, there appears to be a very good agreement between the data of Fu *et al. *(2017) and our results. Of the 22 trinucleotides that allowed protection from phage infection when associated with protospacers, 17 fall into the interfering PAMs group identified in our work. Four protective trinucleotides belong to the intermediate group. One protective trinucleotide of Fu *et al.*, GAT, behaves as stable with either G8 or SP8 protospacers. Of the 42 trinucleotides unable to protect cells from phage infection, 35 belong to the stable group according to our analysis. The remaining seven fall into the intermediate group.

In addition to assaying different PAMs for interference we assessed their ability to support primed adaptation, something that was never addressed before. The primed adaptation mechanism allows the type I CRISPR‐Cas systems to rapidly adjust to accumulation of escape mutations in mobile genetic elements and is thus beneficial to cells. Two contrasting models of primed adaptation have been proposed. According to the first model, CRISPR effector forms structurally different complexes on matched targets with consensus PAMs and targets with nonconsensus PAMs and/or mismatches between crRNA spacer and protospacer. Complexes with the former targets recruit Cas3 that leads to target degradation/interference, while complexes with the later targets recruit Cas3 and Cas1/Cas2 and stimulate acquisition of spacers from imperfect targets (Blosser *et al.*, 2015; Redding *et al.*, 2015). The second model considers primed adaptation to be a consequence of ongoing interference, with overall yield of acquired spacers primarily depending on the kinetics of target degradation (Semenova *et al.*, 2016; Severinov *et al.*, 2016). The results of systematic analysis performed in this work show that PAM variants that abolish CRISPR interference also abolish priming to background levels. These results show that at least some level of interference against foreign DNA is needed to generate material for new spacers and is thus more consistent with the kinetic, interference‐driven model of priming, since the other model posits that it should be possible to achieve primed adaptation without interference. Of 17 PAM variants that support rapid interference, 6 cause negligible adaptation, a result expected for both models. Within the 11 PAM variants that support intermediate levels of interference that allow plasmids to persist over extended periods of time in induced cells, 6 exhibit the highest adaptation score, consistent with kinetic model expectations. The remaining 16 interfering and intermediate PAMs support comparable levels of adaptation. This behavior appears to be inconsistent with simple kinetic model predictions. It is thus possible that effector complexes with targets containing some PAMs from this group, e.g., ATC and CAG, have an increased capacity to attract the Cas1–Cas2 adaptation complex to increase the rate of spacer adaptation before the target is destroyed. The TAT PAM, which causes slow interference, leads to unexpectedly low adaptation yields, again inconsistent with kinetic model. Following the same logic, one may suggest that effector complexes formed on this target are somehow impeded in Cas1–Cas2 interaction.

It has been previously noted that during naïve adaptation a large number of spacers is acquired from protospacers with poorly interfering PAMs (Yosef *et al.*, 2012) or may be ‘slipped’ off‐register with respect to consensus PAM of a protospacer they originate from leading to recognition of sequences with suboptimal PAMs (Shmakov *et al.*, 2014). Our data suggest that such poorly interfering spacers can be highly efficient in promoting primed adaptation which will lead to establishment of high level of resistance to mobile genetic elements. A very recent report from the Fineran group corroborates this idea (Jackson *et al.*, 2019).

Our serendipitous finding that priming (and interference) behavior can be affected by off‐register located consensus PAM sequences shows that the ultimate outcome of priming target recognition can be influenced by the local context outside the standard position of PAM. The recognition of such ‘ectopic’ PAM and/or PAM‐like sequences located close to priming protospacers by the Cse1 subunit of the Cascade may be responsible for formation of distinct conformations of the effector complex with different functional properties that were observed in biophysical experiments (Xue *et al.*, 2016; Krivoy *et al.*, 2018).

**Figure 5:**
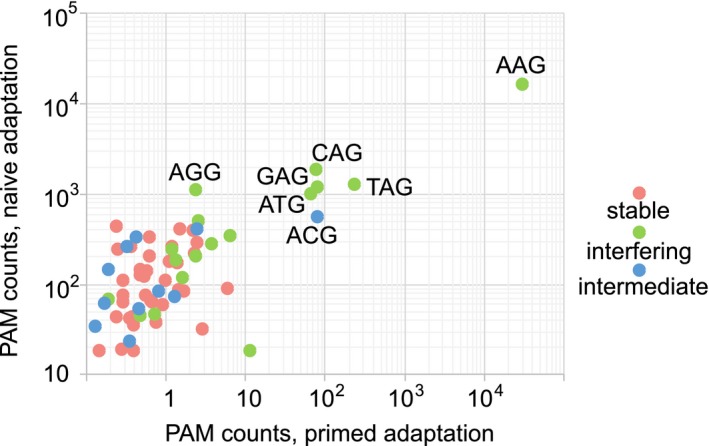
Comparative representation of trinucleotide usage in CRISPR interference, naïve and primed adaptation. The frequencies of trinucleotides corresponding to spacers acquired during naïve adaptation were plotted against their frequencies during primed adaptation in log10 scale. Colors of dots correspond to the three CRISPR interference groups – stable, intermediate or interfering – to which a trinucleotide belongs when attached to a priming protospacer. [Colour figure can be viewed at https://wileyonlinelibrary.com]

The stable nonfunctional PAMs presumably are not recognized by the Cse1 subunit of the Cascade effector. The fact that almost one third of possible trinucleotides support at least some level of interference indicates that Cse1 is capable of recognizing multiple sequences. It thus appears that consensus PAMs revealed by bioinformatics analysis of natural spacer–protospacer matches are due not to specificity of the interference machinery but are instead determined by the adaptation process. Two modes of adaptation characterized in *E. coli*, naïve and primed, have been described. More than 90% of spacers acquired during primed adaptation are derived from protospacers associated with AAG PAM. Less than 50% of spacers acquired in the course of naïve adaptation come from protospacers with AAG. In Fig. 5, we present the frequencies of various trinucleotides associated with protospacers from which Type I‐E spacers are selected during primed or naïve adaptation in *E. coli* (Musharova *et al.*, 2018). To access the efficiency of use of protospacers without the domineering AAG PAM, Fig. 5B shows the frequency distribution for various PAMs without the AAG. Several conclusions can be drawn from this analysis. First, less than 1% of spacers acquired during primed adaptation are associated with stable PAMs and are therefore nonfunctional in interference. In the case of the naïve adaptation, spacers originating from protospacers with nonfunctional PAMs constitute 14%, indicating that even in the absence of priming most acquired spacers are interference‐proficient, likely a consequence of coevolution of the adaptation and interference modules. The second conclusion is that five non‐AAG protospacers most frequently selected during naïve adaptation were associated with CAG, GAG, TAG, AGG, ATG, which belong to the interfering group and this will be functional in defense. Within this group, the ATG was considered, along with AAG, a consensus PAM according to early bioinformatics analysis of protospacers matching natural *E. coli* spacers. The fact that this sequence is not particularly common in protospacers used either during naïve or primed adaptation is likely explained by the presence of a Cas1–Cas2 variants that are distinct from Cas1–Cas2 of K12 *E. coli* used in this work (Mojica *et al.*, 2009). The differences in spacers acquired by such Cas1–Cas2 variants are not expected to have a significant effect on the interference efficiency, since, as our data show, PAM requirements for interference are relaxed.

## Experimental procedures

### Bacterial strains

*E. coli* strains KD471 (K12 F+, *lac*UV5‐*cas3*, *ara*Bp8*‐cse1*, Δ*cas 1,2,* CRISPR I: Repeat‐g8 spacer‐Repeat, ΔCRISPR II + III), KD615 (K12 F+, *lac*UV5‐*cas3*, *ara*Bp8*‐cse1*, CRISPR I: Repeat‐sp8 spacer‐Repeat, ΔCRISPR II + III), KD635 (K12 F+, *lac*UV5‐*cas3*, *ara*Bp8*‐cse1*, Δ*cas 1,2,* CRISPR I: Repeat‐sp8 spacer‐Repeat, ΔCRISPR II + III) were obtained using the previously described recombineering method (Datsenko and Wanner, 2000). *E. coli* KD263 (K12 F+, *lac*UV5‐cas3 *ara*Bp8‐cse1, CRISPR I: Repeat‐g8 spacer‐Repeat, ΔCRISPR II + III) has been described (Shmakov *et al.*, 2014).

### Libraries preparation

To create a pool of plasmids with randomized PAM sequences, PCR products were cloned into a pT7Blue (Novagen) derivate, pWURX1. An *NcoI* restriction site was inserted downstream of the the β‐lactamase ampicillin resistance *bla* gene by PCR using primers BG6629 and BG6630 (Supporting Table S1) and subsequent self‐circularization. The kanamycin resistance gene was amplified from pRSFDuet‐1 (Novagen) plasmid using primers containing a PAM‐protospacer sequence and additional *EcoR*I/*Nco*I restriction sites (Supporting Table S1). Degenerate sequences contain 25% of A, G, C or T at each position. PCR products and pWURX1 were restricted using *EcoR*I and *Nco*I followed by ligation of the PCR products into the plasmid. Resulting pG8‐Km and pSP8‐Km libraries were transformed in ElectroMAX DH5a competent cells. For each library, several thousand colonies growing on selective LB agar plates were pooled and DNA libraries were prepared by plasmid extraction using a miniprep kit (Thermo Scientific).

### High‐throughput plasmid loss experiments

Plasmid libraries were electroporated into KD471 (pG8‐Km based library) or KD635 (pSP8‐Km library) cells. Cells were plated onto LB agar with 50 μg ml^−1^ kanamycin and incubated overnight at 37 °C. Approximately, 40,000–50,000 colonies for each PAM library (~700‐fold excess over the estimated library size) were pooled and resuspended in 10 ml LB without kanamycin. The OD_600_ of cell suspension was adjusted to ~6, and 100 μl of each culture was used to inoculate 150 ml LB without antibiotic and divided into three separate cultures that served as technical replicas. Cells were grown at 37 °C with shaking at 250 rpm until OD_600_ reached 0.4–0.5, and each individual culture was divided into two. Control cultures (CRISPR OFF) continued to grow without *cas* genes induction; in experimental (CRISPR ON) cultures *cas* genes was induced by the addition of 1 mM IPTG and 1 mM arabinose. Two milliliter aliquots were withdrawn prior to the addition of inducers (T1), and 0.5 (T2), 1 (T3), 3 (T4), 6 (T5) and 24 (T6) hours post‐induction. Plasmid DNA was isolated from culture aliquots using GeneJET Plasmid Miniprep Kit (Thermo Scientific). Fragments containing G8 or SP8 protospacer and flanking sequences were amplified with HS Taq DNA polymerase (Evrogen) and Seq_Lib For and Seq_Lib Rev primers (Supporting Table S1). The 98 bp amplicons were purified from agarose gels using GeneJET Gel Purification Kit (Thermo Scientific) and sequenced on MiniSeq Illumina in pair‐end 75 bp long reads mode according to manufacturer’s protocols.

### Analysis of next‐generation sequence data

Raw NGS data were initially inspected using FastQC (v0.11.4) (Shmakov *et al.*, 2014). After trimming using cutadapt (v1.9.1) (Martin, 2011) to remove reads with quality lower than 32, adapters were removed, and only reads longer than 30 bp were used for further analysis. Filtered reads were mapped to pG8‐Km (or pSP8‐Km) plasmid sequence using bwa (ref5961611) (Li and Durbin, 2010). Only matches with quality not less than 10 were kept and converted to bam using samtools (v1.3) (Li *et al.*, 2009). Regions corresponding to PAM and protospacer parts were extracted using pysam (v0.8.4) based Python script. The presence of intact protospacer was checked and only reads fully covering (with no end gaps) the PAM and protospacer regions were selected for further frequency counting using awk. Further analysis was performed using R. For each sample the normalized frequency of PAM occurrences was calculated for all time points using frequencies from corresponding control CRISPR OFF sample and initial (time T1) frequencies (See Supporting Fig. S1 for details). Logfold of frequency changes was calculated by dividing frequency of each PAM by corresponding initial frequency and taking log2 out of this value.

$$\log2{\mathrm{fold}}^{\mathrm{PAM}}\left(t_{i}\right)=\log_{2}\left(\frac{f_{t_{i}}^{\mathrm{PAM}}}{f_{t_{0}}^{\mathrm{PAM}}}\right)$$

Numerical derivative of logfold change was next calculated.

$$\frac{d\left[{\log2\mathrm{fold}}^{\mathrm{PAM}}\left(t_{i}\right)\right]}{\mathrm{dt}}=\frac{{\log2\mathrm{fold}}^{\mathrm{PAM}}\left(t_{i}\right)-{\log2\mathrm{fold}}^{\mathrm{PAM}}\left(t_{i-1}\right)}{t_{i}-t_{i-1}}$$

Clustering of time series based on numerical derivatives was performed using *pvclust* package (v.2.0‐0) (Suzuki and Shimodaira, 2006), with 2000 iterations.

### HTS data normalization

Because of active interference process, frequencies of some PAMs could increase due to the depletion of others even though the actual amount of cells carrying plasmids with such PAMs is unchanged. In order to exclude the influence of such effects on our analysis, the following empirical normalization technique was used. For each time point, frequencies of depleted PAMs (those whose frequency was

$$f_{i}^{\mathrm{corrected}}=f_{i}\left(1-\sum_{j:f_{j}<0.5/64}f_{j}\right)$$

less than 0.5/64 = 1/128) were summed. Then all frequencies for this time point were scaled by factor 1 minus such sum. Examples of corrected series frequencies for several representative PAMs could be found on Supporting Fig. S1B. When correcting raw read counts for each time point, the data in CRISPR ON experiments (total number of counts for all PAMs) were adjusted to be equal to that in CRISPR OFF controls, and then counts for each PAM were scaled by ratio of corrected to raw frequencies (fcorrected/fraw) for the same PAM. The effect of our rescaling procedure on all PAMs could be seen from Supporting Fig. S1. After correction, the PAMs unaffected by interference tend to be closer to the red diagonal line, showing no difference between experimental and control cultures.

### Generation of individual PAM variant plasmids

KD263 cells transformed with pG8‐Km PAM library were plated on selective medium. Individual colonies were subjected to PCR with primers amplifying the PAM‐G8 protospacer region. The PCR products were analyzed by Sanger sequencing and colonies with all 64 possible PAM variants were selected one after another.

Plasmids pGAC‐SP8, pCAA‐SP8 and pCTG‐SP8 are pAAG‐SP8 derivatives containing GAC, CAA or CTG PAM instead of AAG PAM in front of the priming protospacer. The mutation was introduced by standard site‐directed mutagenesis protocol with primers IP_SP8 for and IP_SP8 rev (Supporting Table S1).

Plasmids pAGA*‐G8, pAGT*‐G8, pAGC*‐G8 and pAGG*‐G8 are pAAG‐G8 derivative containing a TCT trinucleotide upstream of the AGN PAM instead of TAA trinucleotide present in pG8‐Km. The mutation was introduced by standard site‐directed mutagenesis protocol with primers IP_G8 for and IP_G8 rev (Supporting Table S1).

### *In vivo* analysis of primed CRISPR adaptation

KD263 and KD615 cells were transformed with individual plasmids containing different PAM variants and, correspondingly, G8 or SP8 protospacer. Single colonies were picked, inoculated in liquid LB containing 50 μg ml^−1^ of kanamycin and grown overnight. Aliquots of cultures were used to inoculate fresh LB without antibiotic and growth at 37°C was allowed for few hours until OD_600_ reached 0.4. Expression of *cas* genes was induced by the addition of 1 mM IPTG and 1 mM arabinose. At various times, 100 μl culture aliquots were withdrawn. 1 μl of withdrawn culture aliquots was used in a 20 μl PCR reaction with Taq polymerase using primers EcLDR For and g8 Rev (for KD263) (or sp8 rev for KD615) to amplify the CRISPR array. The PCR product of unexpanded CRISPR array is 118 bp; a product of amplification of CRISPR array expanded by one spacer‐repeat unit is 179 bp. PCR products were analyzed on 2% agarose gels and gel images were quantified using Image Lab 5.0 software. Approximately 100 ng of purified DNA amplicons for several randomly selected PAM variant were sequenced using Illumina MiniSeq system according to the recommended protocol of the manufacturer. Results were analyzed as described earlier (Semenova *et al.*, 2015).

The efficiency of primed adaptation was measured using qPCR as described earlier (Krivoy *et al.*, 2018). The amount of CRISPR arrays that acquired a particular plasmid‐derived spacer (hot spot 1 (HS1 5′ GCTTTCCCTATAGTGAGTCGTATTAGAGCTTGG 3′), hot spot 2 (HS2 5′ GAGTATGAGCCATATTCAACGGGAAACGTCTTG 3′) and hot spot 3 (HS3 5′ GAGTTGGTAGCTCTTGATCCGGCAAACAAACCA 3′) was quantified and normalized by the amount of the *gyrA* gene from the bacterial genome (Supporting Fig. S4).

The qPCR scores corresponding to HS1, HS2, HS3 were normalized for the score obtained with CAA PAM plasmid and were next made equal for all PAM plasmids in all replicas:

$${normalized\;score}_{{\mathrm{HS}}_{i}}^{\mathrm{PAM}}={\mathrm{score}}_{{\mathrm{HS}}_{i}}^{\mathrm{PAM}}\frac{{\mathrm{score}}_{HS_{1}}^{\mathrm{CAA}}}{{\mathrm{score}}_{HS_{i}}^{\mathrm{CAA}}},\;i=2,\;3$$

Normalized scores for each hot spot were averaged between hotspots and measurements. The Spearman rank correlation for raw and normalized and averaged data was high, see Supporting Table S2.

Since the CAA PAM score was not available for experiments with the special subset of PAMs (AGN*, see the Results section for details), a separate normalization procedure was used. We normalized the hotspot scores using AGC PAM HS1 as a benchmark. The averaging itself was done similar to the general case described above. However, to rank the special PAMs with the general set of PAMs, we normalized their scores by the score of the AGC, PAM which was present in both plasmid contexts (see Results). To do so, we multiplied the averaged ‘special’ scores ${\mathrm{score}}^{AGN*}$ by the fraction of scores for AGC PAM in the general and special sets (see Fig. 4A and D).

$$\left\langle {rescaled\;score}^{AGN*}\right\rangle \;=\;\left\langle {\mathrm{score}}^{AGN*}\right\rangle \;\frac{\left\langle {\mathrm{score}}^{\mathrm{AGC}}\right\rangle }{\left\langle {\mathrm{score}}^{AGC*}\right\rangle },\;N=A,\;C,\;T,\;G$$

The obtained $\left\langle {rescaled\;score}^{AGN*}\right\rangle$ were placed in the common table completing Fig. 4A.

Datasets of spacers acquired from plasmid pG8_Km in courses of naïve or primed adaptation were adopted from Musharova *et al.* (2018) in order to compare PAM usage frequency. All spacers were assigned according corresponding PAM motifs, and the mean of quantities between experimental replicates for each trinucleotide motif was calculated and plotted.

## Conflict of interest

The authors declares that there is no conflict of interest regarding the publication of this article.

## Author contributions

K.S. and S.B. devised the study. O.M. designed and performed the experiments; V.S. designed and performed all bioinformatical procedures; M.V. cloned libraries; K.D. designed *E. coli* strains; E.S. and E.S. revised the manuscript critically; A.K. and I.F. designed and analyzed pPCR experiments. All authors approved the final version of the manuscript.
