# Supplementary material for: Systematic analysis of Type I‐E Escherichia coli CRISPR‐Cas PAM sequences ability to promote interference and primed adaptation
Source: Mol Microbiol. 2019 Apr 6;111(6):1558–70. doi: 10.1111/mmi.14237 (PMC6568314; doi:10.1111/mmi.14237)
Supplement: Supplementary file 1 [file MMI-111-1558-s001.docx]

**SUPPLEMENTARY DATA**

| **Name** | | **Sequence 5’—3’** |
| --- | --- | --- |
| **HTS analysis** | | |
| **Seq_Lib For** | | GTTTCATTTGATGCTCGATGAG |
| **Seq_Lib Rev** | | ATATCGGATCCCCGGGTA |
| **CRISPR array elongation analysis** | | |
| **EcLDR For** | | AAGGTTGGTGTCTTTTTTAC |
| **G8_Rev** | | GTCGCTGCCGTGACGTTATG |
| **SP8_Rev** | | AAAAGTGCCACTTGCGGAGAC |
| **qPCR** | | |
| **HS1_Rev** | | CCAAGCTCTAATACGAC |
| **HS2_Rev** | | TTGTTTGCCGGATCAAGA |
| **HS3_Rev** | | CAAGACGTTTCCCGTTGA |
| **Cloning** | | |
| **IP_GAC_SP8_For** | | TGAGTTTTTCTAAGACCTGACGACC |
| **IP_GAC_SP8_Rev** | | GGT CGT CAGCTGTTAGAAAAACTCA |
| **IP_CAA_SP8_For** | | TGAGTTTTTCTAACCGCTGACGACC |
| **IP_CAA_SP8_Rev** | | GGTCGTCAGGGCTTAGAAAAACTCA |
| **IP_CTG_SP8_For** | | TGAGTTTTTCTAACAACTGACGACC |
| **IP_CTG_SP8_Rev** | | GGTCGTCAGTTGTTAGAAAAACTCA |
| **IP_CCG_SP8_For** | | TGAGTTTTTCTAACTGCTGACGACC |
| **IP_CCG_SP8_Rev** | | GGTCGTCAGCAGTTAGAAAAACTCA |
| **IP_AGA*_G8_For** | | AGACTGTCTTTCGCTGCTGAGGGTG |
| **IP_AGT*_G8_For** | | AGTCTGTCTTTCGCTGCTGAGGGTG |
| **IP_AGC*_G8_For** | | AGCCTGTCTTTCGCTGCTGAGGGTG |
| **IP_AGG*_G8_For** | | AGGCTGTCTTTCGCTGCTGAGGGTG |
| **IP_AGN*_G8_Rev** | | AGAAAAACTCATCGAGCATCAAATG |
| **Library preparation*** | | |
| **BG6629** | | CAAGTTTACTCATATATACTTTAGATTG |
| **BG6630** | | CCATGGGTCTGACAGTTACCAATGCTT |
| **BG6385** | Reverse primer | ATCCTTTAAACCATGGGCGCTGCATGCCTATTTG |
| **BG6548** | SP8 based Library | **AAG**CTGACGACCGGGTCTCCGCAAGTGGCACTTTT |
| **BG6550** | G8 based Library | **AAG**CTGTCTTTCGCTGCTGAGGGTGACGATCCCGC |

**Supplementary Table S1. Primers used in this work.**

***** Degenerate sequences are in bold

**a)** **HS1-CAA normalized**

|  | **HS1** | **HS1_2** | **HS2** | **HS3** |
| --- | --- | --- | --- | --- |
| **HS1** | 1.00 | 0.91 | 0.81 | 0.85 |
| **HS1_2** | 0.91 | 1.00 | 0.87 | 0.90 |
| **HS2** | 0.81 | 0.87 | 1.00 | 0.91 |
| **HS3** | 0.85 | 0.90 | 0.91 | 1.00 |

**b) Raw**

|  | **HS1** | **HS1_2** | **HS2** | **HS3** |
| --- | --- | --- | --- | --- |
| **HS1** | 1.00 | 0.90 | 0.79 | 0.83 |
| **HS1_2** | 0.90 | 1.00 | 0.86 | 0.89 |
| **HS2** | 0.79 | 0.86 | 1.00 | 0.89 |
| **HS3** | 0.83 | 0.89 | 0.89 | 1.00 |

**Supplementary Table S2. The Spearman's *rho* for the G8 protospacer library adaptation scores.**

**Supplementary Figure S1.**

**
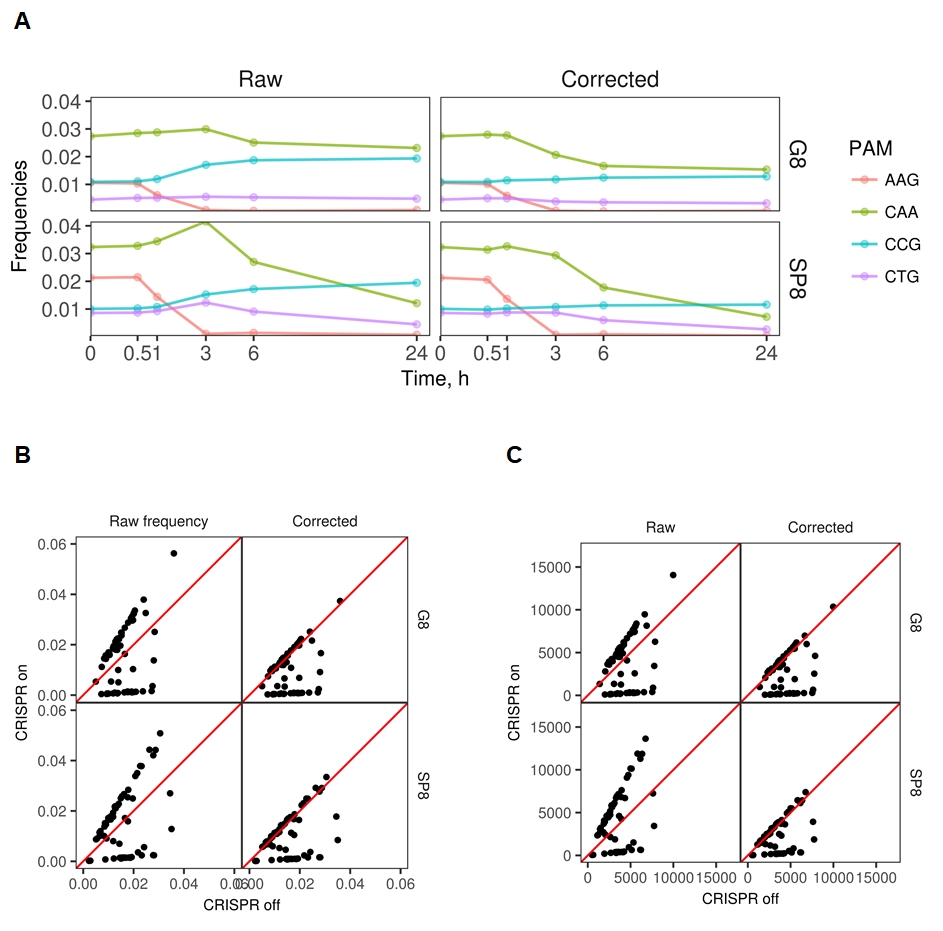
**

**A.** Raw and corrected frequencies for several indicated PAMs in cultures transformed with SP8 and G8 libraries.

**B.** PAM frequencies for CRISPR ON and CRISPR OFF samples for G8 and SP8 protospacer libraries at 6 hour time point. The red line corresponds to frequency ratio of 1, or no difference between control and experimental conditions. Each dot corresponds to an individual PAM variant.

**C.** PAM counts (individual reads) for CRISPR ON and CRISPR OFF samples of the SP8 protospacer library at 6 hour time point.

**Supplementary Figure S2.**

**A.** Dynamics of PAM loss in SP8 and G8 protospacer libraries shown as a violin-plot movie.

**B.** A movie illustrating PAM loss in SP8 and G8 libraries based on Fig. 2 of the main text. Each dot shows a frequency of an individual PAM variant under CRISPR ON (vertical axis) and CRISPR OFF (horizontal axis) conditions. Red, green, and blue dots indicate, respectively, stable, interfering and intermediate group variants.

**Supplementary Figure S3.**

**
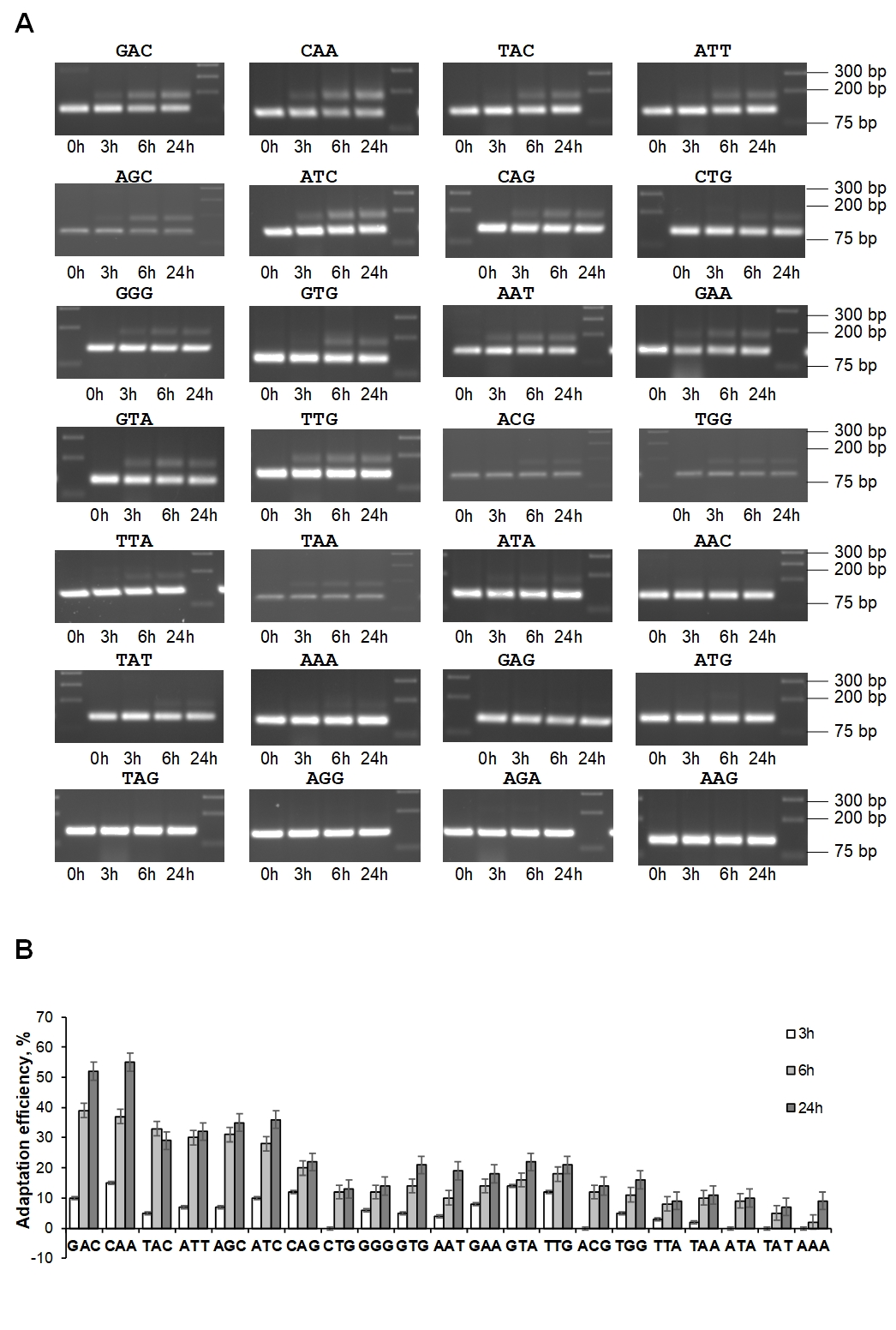
**

**C**


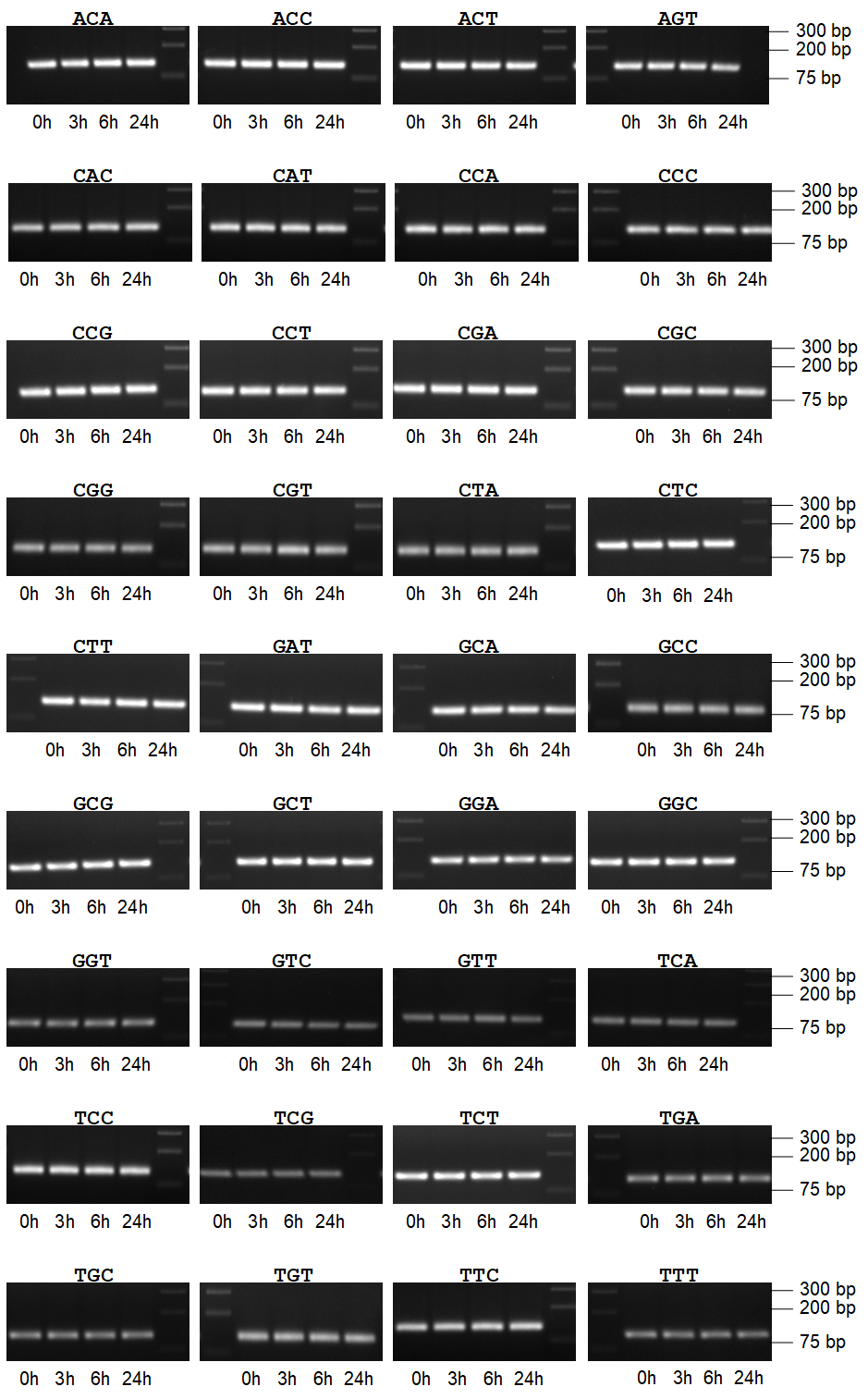


**A.** Expansion of CRISPR arrays in KD263 cultures transformed with G8 protospacer plasmids with indicated PAM variants.

**B.** Quantification of adaptation efficiency based on relative intensities of ethidium bromide staining of bands corresponding to expanded and initial CRISPR array amplicons.

**C.** Expansion of CRISPR arrays in KD263 cultures transformed with G8 protospacer plasmids with stable PAM variants

**Supplementary Figure S4.**

**
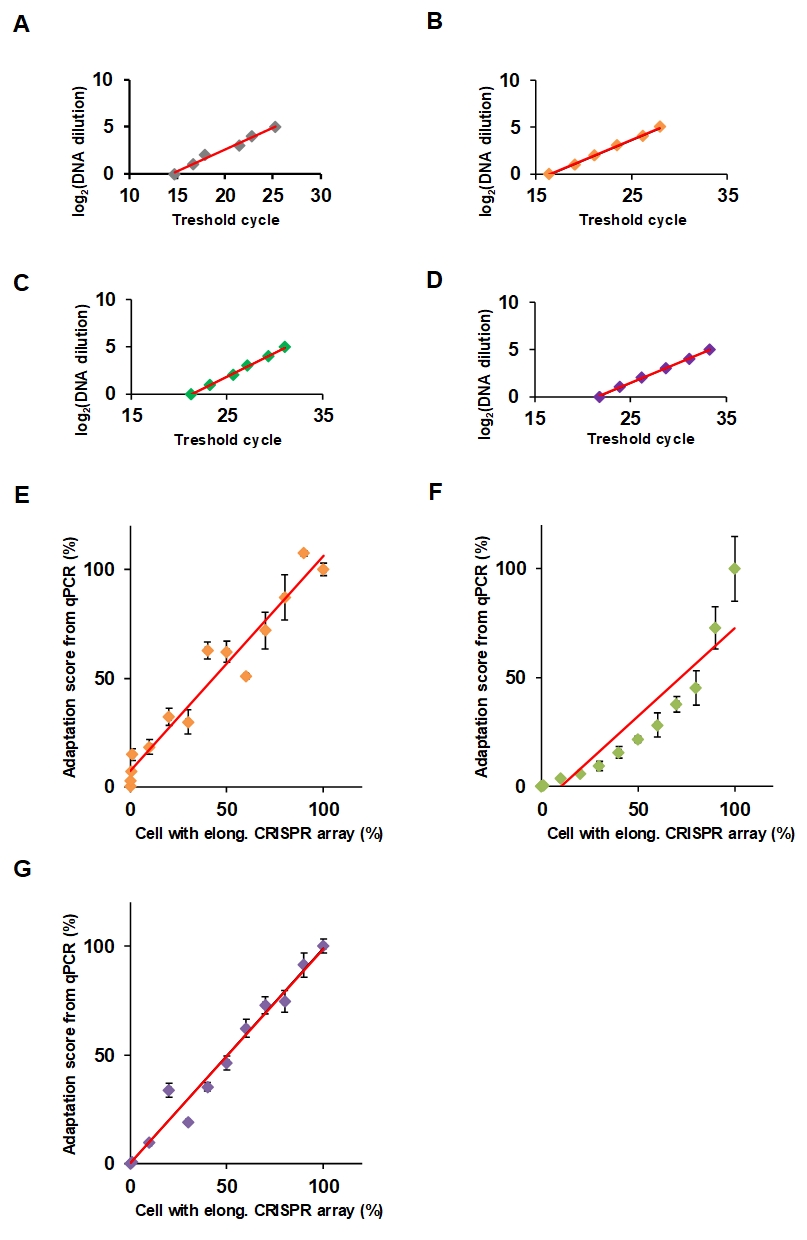
**

**A.** Calibration of qPCR measurements on the *gyrA* gene used for normalization. Genomic DNA from cells containing an elongated CRISPR array with HS1 was diluted between 1- and 4096-fold and subjected to qPCR to determine the threshold cycle. Plotting the binary (base-2) logarithm of the DNA dilution over the threshold cycle value provides the expected linear relation with a slope of 0.978 (red line, R² = 0,9856) corresponding to a 1.97-fold DNA increase per PCR cycle.

**B, C,** and **D.** Calibration of qPCR measurements on HS1 (**B**), HS2 (**C**), HS3 (**D**) amplicons. Genomic DNA of KD263 cells derivatives containing CRISPR arrays expanded with HS1, HS2, or HS3 spacers was diluted between 1- and 4096-fold and subjected to qPCR to determine the threshold cycle. The slope of a linear fit to the data is 0.4224 (HS1), 0.49 (HS2) and 0.429 (HS3) corresponding to a 1.796-fold DNA increase per PCR cycle (HS1), 1.972-fold DNA increase per PCR cycle (HS2) and 1.812-fold DNA increase per PCR cycle (HS3).

**E, F,** and **G.** Testing qPCR-based quantification of primed adaptation. KD263 cells containing either a non-expanded CRISPR array or CRISPR arrays expanded with HS1 (**E**), HS2 (**F**), or HS3 (**G**) spacers were mixed at indicated ratios and genomic DNA was extracted and subjected to qPCR analysis. The adaptation score was obtained from the ratio of the amount of DNA containing elongated CRISPR arrays and the amount of DNA containing the *gyrA* gene. The DNA amounts were calculated from measured qPCR threshold cycles using the calibration curves presented in earlier panels of the figure. The measured adaptation score returns within error the input percentage of cells containing CRISPR arrays expanded by each of the three hot spot spacers, thus verifying the calibration.

**Supplementary Figure S5.**

**
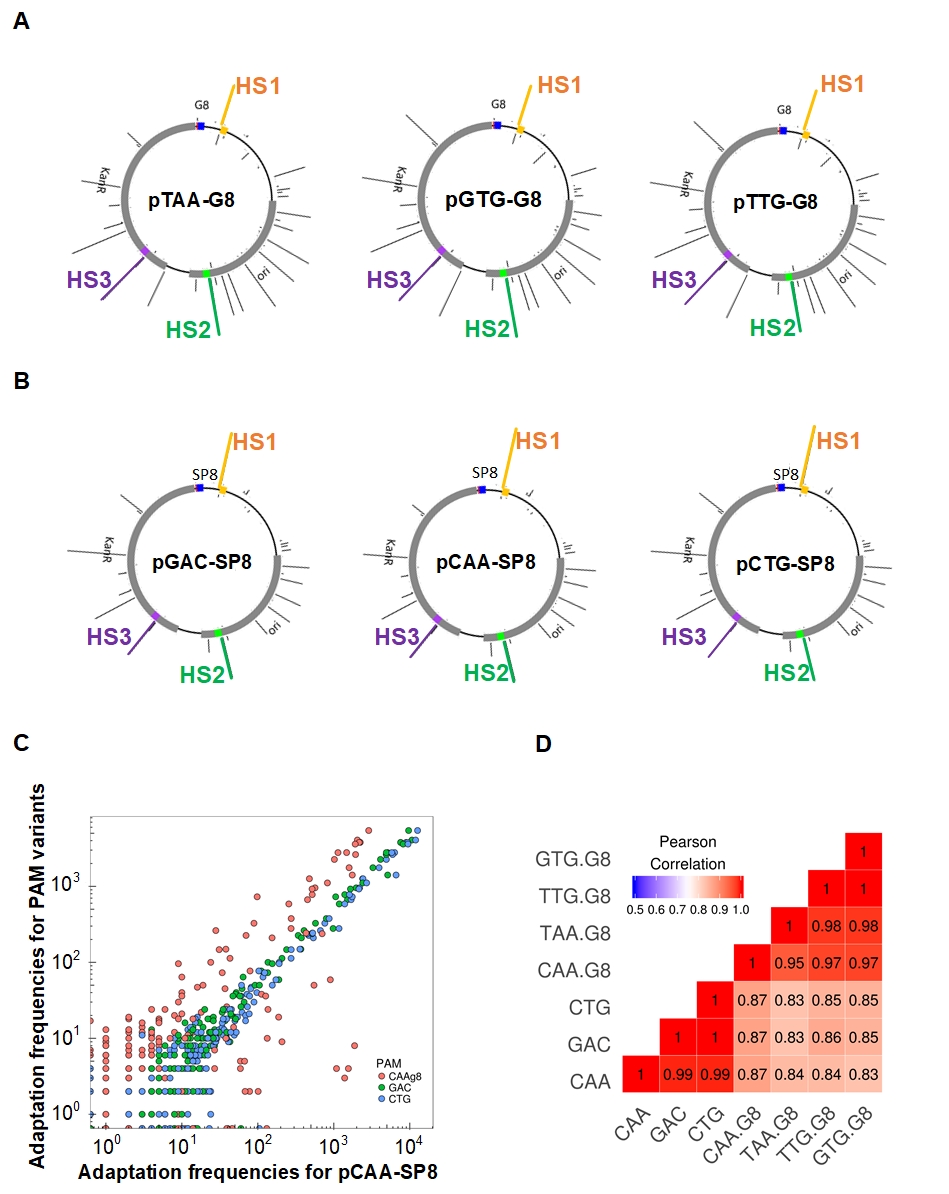
**

**A.** Mapping of spacers acquired from the pTAA-G8, pGTG-G8 and pTTG-G8 PAM variant plasmids to the pT7blue-Km backbone. The heights of bars correspond to the number of HTS reads found for a particular protospacer position. The priming protospacer and its PAM are shown as blue and red boxes. Bars marked orange, green, and purple, indicate, respectively, hotspots HS1, HS2 and HS3, which were used for qPCR analysis and normalizations.

**B.** Mapping of spacers acquired from the pGAC-SP8, pCAA-SP8 and pCTG-SP8 PAM variants plasmid to the pT7blue-Km backbone. See legend for panel **A** for details.

**C.** Position-dependent acquisition frequency for pGAC-SP8, pCTG-SP8 PAM variants and pCAA-G8 plasmid plotted over the acquisition frequency for the pCAA-SP8 plasmid. High correlation between spacer acquisition patterns of all tested PAM variants is apparent.

**D.** Pearson correlation coefficients of position-dependent spacer acquisition frequencies for indicared PAM variants in SP8 and G8 protospacer-containing plasmids. Acquisition frequencies are highly correlated to each other (correlation coefficients of >0.83 between different protospacer plasmids, and >0.95 between plasmids with same protospacer).

**Supplementary Figure S6.**

**
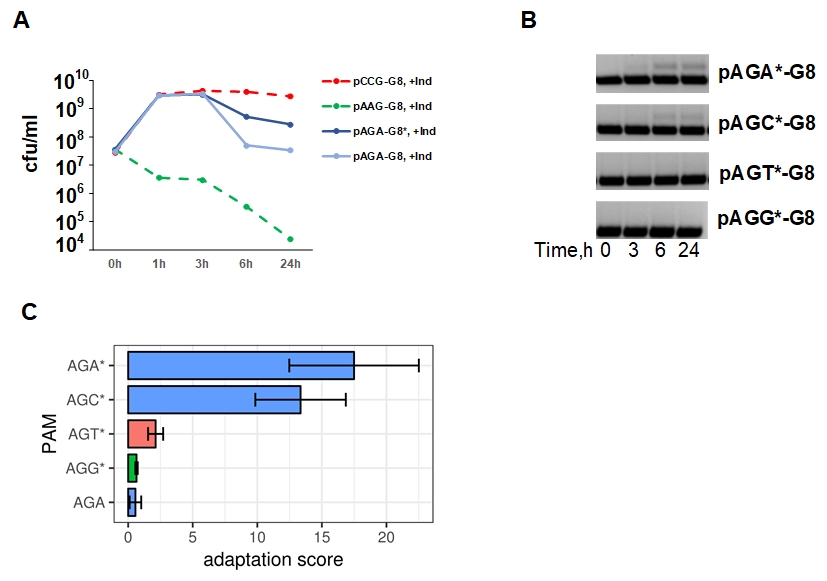
**

KD263 cells were transformed with indicated G8 protospacer PAM variants. Asterisks indicate altered context of AGA PAM (see Fig. 4B). Plasmids carrying CCG and AAG PAMs were used as controls. In **A**, the number of kanamycin-resistant CFUs in cultures transformed with each plasmid at indicated times after the induction of *cas* gene expression is shown. In **B**, agarose gels showing expansion of CRISPR arrays in cultures analyzed transformed with AGN PAMs plasmid with altered upstream context are presented. **C.** The adaptation score obtained for the AGN* PAM variants.
