# Supplementary figures and images for: Systematic analysis of Type I‐E Escherichia coli CRISPR‐Cas PAM sequences ability to promote interference and primed adaptation
Source: Mol Microbiol. 2019 Apr 6;111(6):1558–70. doi: 10.1111/mmi.14237 (PMC6568314; doi:10.1111/mmi.14237)

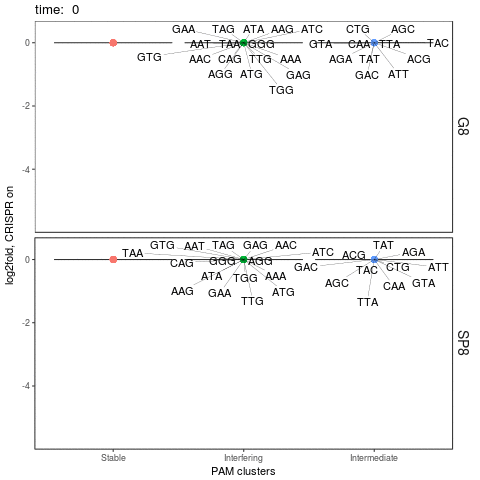

Supplement: Supplementary file 2 [file MMI-111-1558-s002.gif]

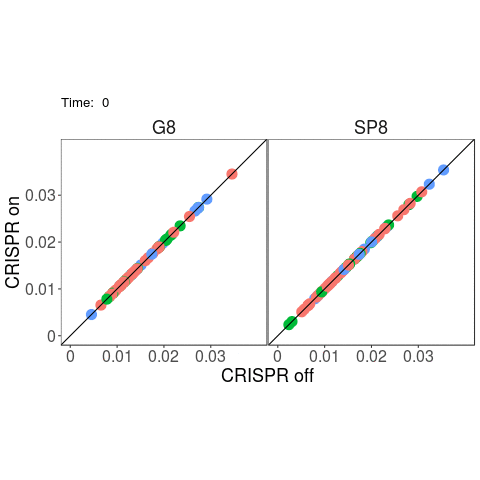

Supplement: Supplementary file 3 [file MMI-111-1558-s003.gif]
